# Can Preening Contribute to Influenza A Virus Infection in Wild Waterbirds?

**DOI:** 10.1371/journal.pone.0011315

**Published:** 2010-06-25

**Authors:** Mauro Delogu, Maria A. De Marco, Livia Di Trani, Elisabetta Raffini, Claudia Cotti, Simona Puzelli, Fabio Ostanello, Robert G. Webster, Antonio Cassone, Isabella Donatelli

**Affiliations:** 1 Department of Veterinary Public Health and Animal Pathology, Faculty of Veterinary Medicine, University of Bologna, Ozzano Emilia, Italy; 2 Department of Infectious, Parasitic and Immune-Mediated Diseases, Istituto Superiore di Sanità, Rome, Italy; 3 Department of Food Safety and Veterinary Public Health, Istituto Superiore di Sanità, Rome, Italy; 4 Istituto Zooprofilattico Sperimentale della Lombardia e dell'Emilia-Romagna, Lugo, Italy; 5 Division of Virology, Department of Infectious Diseases, St. Jude Children's Research Hospital, Memphis, Tennessee, United States of America; University of Georgia, United States of America

## Abstract

Wild aquatic birds in the Orders Anseriformes and Charadriiformes are the main reservoir hosts perpetuating the genetic pool of all influenza A viruses, including pandemic viruses. High viral loads in feces of infected birds permit a fecal-oral route of transmission. Numerous studies have reported the isolation of avian influenza viruses (AIVs) from surface water at aquatic bird habitats. These isolations indicate aquatic environments have an important role in the transmission of AIV among wild aquatic birds. However, the progressive dilution of infectious feces in water could decrease the likelihood of virus/host interactions. To evaluate whether alternate mechanisms facilitate AIV transmission in aquatic bird populations, we investigated whether the preen oil gland secretions by which all aquatic birds make their feathers waterproof could support a natural mechanism that concentrates AIVs from water onto birds' bodies, thus, representing a possible source of infection by preening activity. We consistently detected both viral RNA and infectious AIVs on swabs of preened feathers of 345 wild mallards by using reverse transcription–polymerase chain reaction (RT-PCR) and virus-isolation (VI) assays. Additionally, in two laboratory experiments using a quantitative real-time (qR) RT-PCR assay, we demonstrated that feather samples (*n* = 5) and cotton swabs (*n* = 24) experimentally impregnated with preen oil, when soaked in AIV-contaminated waters, attracted and concentrated AIVs on their surfaces. The data presented herein provide information that expands our understanding of AIV ecology in the wild bird reservoir system.

## Introduction

Of the numerous wild bird species susceptible to avian influenza viruses (AIVs), species in the Orders Anseriformes and Charadriiformes are the main known reservoir hosts that can perpetuate the genetic pool of influenza A viruses [1]. The co-evolution of the host/pathogen system has favored, by natural selection, a well-adapted bird/virus relationship in which low-pathogenicity AIVs (LPAIVs) cause asymptomatic infections in which virus preferentially replicates in the gastrointestinal tract of reservoir hosts [2]. However, occasional transmissions to poultry species can generate high-pathogenic avian influenza viruses (HPAIVs) such as the Eurasian lineage H5N1 HPAIV, which has important implications for both public and veterinary health [3],[4]. Viral replication in the intestines leads to high viral loads in the feces of aquatic birds and on natural habitats, such as wetlands, the fecal–oral route is believed to be an efficient route of transmission [5],[6]. The role of aquatic environments in the transmission of AIVs in nature has been extensively studied (reviewed in [7]), and AIVs were recently detected in sediments of ponds by using molecular methods [8]. Although surface water is a source from which AIVs have been isolated, fecal shedding in water may result in progressive dilution of the viral load, gradually decreasing the likelihood of interaction between AIVs and hosts.

Laboratory methods to concentrate virus have been developed to recover AIVs from environmental water samples [9],[10]. Whether similar natural mechanisms that concentrate AIVs from water onto bird bodies exist remains unknown. Furthermore, no mechanisms to counteract potential viral dilution in water have been identified. In such a context, there may be a missing ecological link between aquatic birds and the environment, which fits into the fecal-water-oral route of transmission.

To address these concerns, we sought a common denominator among aquatic birds that could attract AIVs from water and, thus, connect different avian taxonomic groups, such as ducks, grebes, loons, gulls, and shorebirds. All aquatic birds, regardless of their epidemiologic roles in influenza ecology [11], waterproof their feathers by the process of preening [12]-[16]. To achieve effective insulation, birds spread preen oil from their uropygial glands, also known as preen oil glands, all over their plumage [17]. Thus, aquatic birds become covered with an invisible, mostly lipidic [12] film that interacts with water, some of which may be contaminated with AIVs shed in the feces of infected birds [18].

We hypothesized that the preen oil acts as a natural capture system causing active and progressive concentration of AIV particles from water onto birds' bodies. To test the feasibility of this proposed mechanism, we conducted two complementary studies: (1) an epidemiologic investigation to determine whether AIVs are present on feather and cloacal swabs of wild mallards; and (2) a laboratory-based trial to determine whether feathers experimentally impregnated with preen oil and subsequently soaked in AIV–contaminated water, could capture and concentrate AIVs.

## Materials and Methods

### Field Studies to Detect and Characterize AIVs

**Figure 1:**
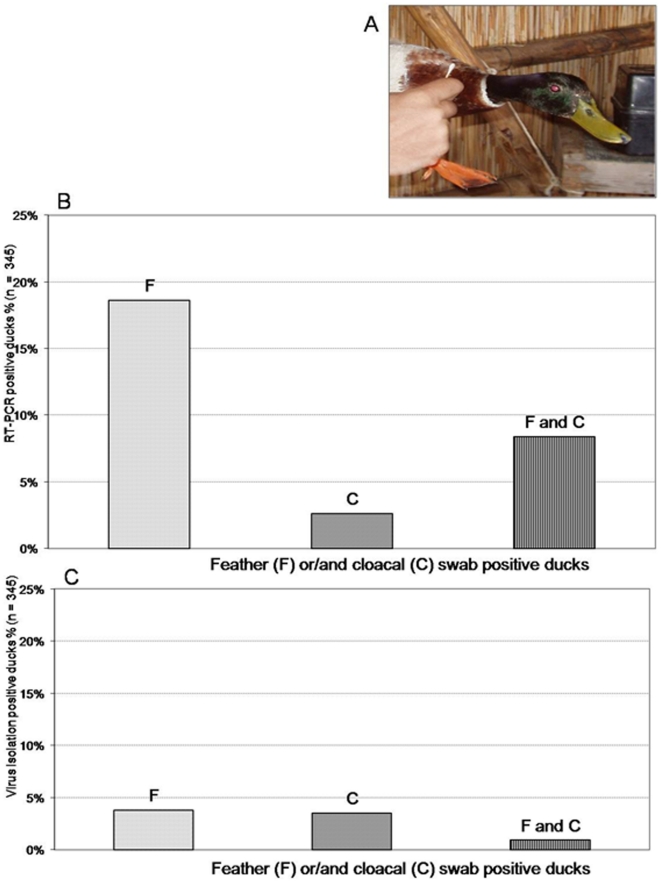
Field Studies: The novel sampling approach and virological results from feather and cloacal swabs taken from wild mallards. (A) Feather swabs were obtained by rubbing feather surface around the waterline level of birds. (B) Molecular results showing percentages of RT-PCR–positive ducks (*n* = 345) for matrix (M) gene of influenza A virus: 64/345 (18.6%) mallards were positive from feathers only (F), 9/345 (2.6%) from cloaca only (C), and 29/345 (8.4%) from both feathers and cloaca (F and C). (C) Influenza A virus isolation (VI) results: 13 ducks (3.8%) were VI-positive from feathers only (F), 12 (3.5%) from cloaca only (C), and 3 (0.9%) from both feathers and cloaca (F and C). Denominator for prevalence calculation is the same as in A (*n* = 345) because, after the initial molecular screening, we assumed that PCR-negative ducks were VI-negative ducks as well.

From December 2006 to August 2007, 345 wild mallards (*Anas platyrhynchos*) were trapped in wetlands of central Italy (Laguna di Orbetello World Wildlife Fund oasis, Tuscany region). The most representative dabbling duck species of the Western Palearctic region was sampled, which is an important natural reservoir of AIVs [19]. A new sampling approach to detect AIVs on birds' body surfaces was developed (Figure 1A). Feathers were sampled by rubbing a cotton swab over an approximately 100 cm^2^ area on the duck feathers located around the bird's waterline level (anatomic areas of breast and flanks were rubbed at least five times with a swab). To exclude possible fecal contamination of feathers during sampling, ducks were captured by cage traps placed in the water and then handled individually. Concurrent with feather swabbing, cloacal swabs were also collected from each duck. Both feather and cloacal swabs were individually placed in 1000 µL transport medium (1∶1 PBS∶glycerol with potassium penicillin, streptomycin sulfate, gentamicin sulfate, polymyxin B, mycostatin) and stored at −80°C until laboratory testing.

To detect AIVs, a highly sensitive PCR-based method [20] was used to initially screen pooled samples of feather and cloacal swabs (Figure S1). Specifically, the presence of influenza A virus particles in the feather swab samples were screened by one-step reverse transcription–polymerase chain reaction (RT-PCR) specific for matrix (M) gene amplification. Briefly, 40 µL of transport medium were collected from each of five feather swabs and pooled in a single tube. Viral RNA (vRNA) was extracted from the pooled sample using the QIAmp Viral RNA MiniKit (Qiagen, GmbH, Hilden, Germany) according to the manufacturer's instructions. The vRNA from each pool was then amplified by the one-step RT-PCR assay using the 1x SuperScript III One-Step RT-PCR system with PlatinumTaq DNA Polymerase (Invitrogen, Grand Island, NY). The 15-µL reaction volume contained 6.6 µL extracted RNA, 1× III One-Step RT-PCR reaction mix, M52C and M253R oligonucleotides [20] (each at a concentration of 20 µM), and SuperScript III RT/Platinum Taq DNA polymerase (2U). Thermocycling was performed in an I-cycler thermal cycler apparatus (Bio-Rad, Hercules, CA) under the following conditions: 30 min at 45°C and 2 min at 94°C once, and then 45 s at 94°C, 45 s at 55°C, and 45 s at 72°C 40 times. This step was followed by 1 cycle of 72°C for 7 min. The resulting PCR products were separated by gel electrophoresis through a 2% (w/v) agarose gel in Tris-acetate buffer, stained with ethidium bromide, and visualized under UV light with a Fluor-S MultiImager quantitative imaging system (Bio-Rad). Cloacal swabs were treated and examined by one-step RT-PCR as described above for the feather swabs (Figure S1). When pooled samples of cloacal or feather swabs were verified to be RT-PCR–positive, each individual sample in that pool were retested by RT-PCR to identify the AIV–positive duck.

To confirm virus infectivity, feather and cloacal swab samples that were positive via RT-PCR were further tested by VI in embryonated chicken eggs (Figure S1). Specific pathogen–free embryonated chicken eggs (9- to 11-days old) were inoculated with clarified transport media from a single RT-PCR–positive sample according to standard procedures [21]. Inoculated allantoic fluid was examined using the hemagglutination (HA) assay [21] and an ELISA test specific for influenza A virus nucleoproteins [22].

Influenza A virus isolates were further characterized with the serologic hemagglutination inhibition (HI) assay [21], RT-PCR [23] and partial sequencing of the HA gene (data not shown).

### Laboratory Experiments to Detect and Quantify AIVs

To simulate natural conditions in the laboratory experiments performed with feathers and swabs, we used water collected in the field to reproduce the water-mediated AIV–preen oil interaction. Among the chemical and physical parameters of water (i.e., salinity, pH, and temperature) that reportedly influence viral persistence [24]–[27], we verified whether salinity could affect the hypothesized AIV–capture mechanism of preened surfaces. Therefore, we used salt water in experiment 1 and fresh water in experiment 2.

In experiment 1, the virus concentrations of AIV-contaminated water and preened feathers soaked in the same water were compared after a 24-h incubation as follows: LPAIV A/Mallard/Italy/228090/2005 (H5N1) (allantoic fluid) was diluted in 2.5 L of salt water (pH 7.4, salinity 45 ppt, density 1045 kg/m^3^, previously tested negative for AIVs) to achieve a final virus concentration of 10^3.9^ EID_50_/mL water.

**Figure 2:**
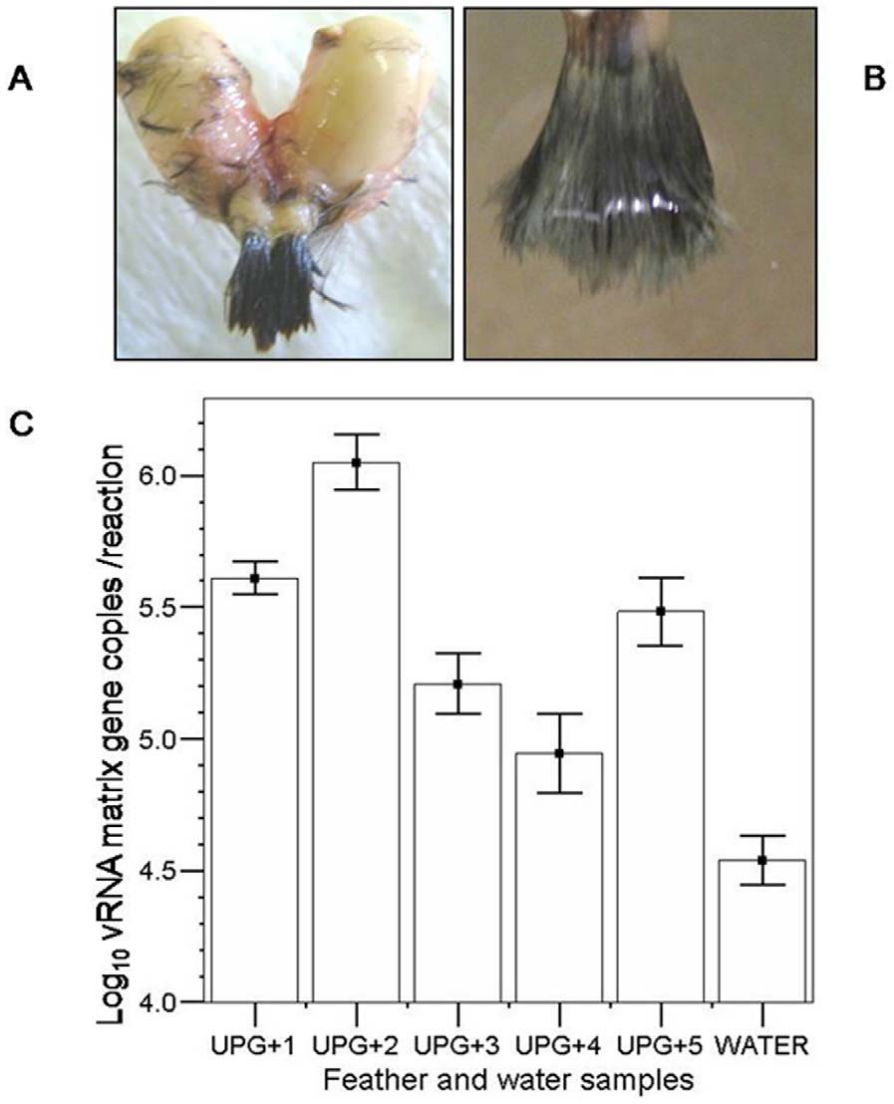
Interactions between preened feather tufts and AIV–contaminated salt water. (A) Feather tufts surrounding the oil gland papilla were taken from slaughtered mallards (*n* = 5) and preened by squeezing the uropygial gland. (B) Preened feather tufts were soaked in AIV-contaminated salt water for 24 h before qRRT-PCR assays to detect the M gene. (C) The mean ±2 SEMs of log_10_ of M gene copies per reaction from each mallard (UPG+1 to UPG+5) is shown; the contaminated salt water was used for comparison.

Five feather tufts surrounding the oil gland papilla (Figure 2A) were collected at the necropsy facilities of the Faculty of Veterinary Medicine (University of Bologna) from slaughtered mallards purchased from a fowl processing station. Each tuft was preened by squeezing the uropygial gland (preen oil and ducks were previously tested negative for AIVs; data not shown) and subsequently soaked in AIV–contaminated water at 10°C (Figure 2B). After 24 h, each feather tuft was removed and incubated in 500 µL transport media for 3 h at 4°C to improve virus release from feather tufts. After vortexing for 5 min, each feather tuft was suspended in a tube and centrifuged for 10 min at 200×g. The final volume of each sample was approximately 600 µL, including approximately 100 µL contaminated water that was adsorbed by the feathers during soaking time. A separate water sample collected at the same water level was used as a control to evaluate AIV concentration in the feather tuft. Processed transport media and water samples were stored at −80°C until examination with the one-step qRRT-PCR assay using a minor groove–binder probe assay as previously described [28].

Viral RNA was extracted from the transport media and water sample (QIAmp Viral RNA MiniKit, Qiagen) and then amplified by qRRT-PCR with primers and a probe targeting a highly conserved region of the M gene of influenza A viruses. The influenza matrix RNA was then transcribed in vitro from the corresponding DNA template, cloned into a plasmid vector as previously described [28], and used as the standard RNA to generate standard curves for quantification of the vRNA in samples.

Briefly, the 25-µL reaction volume contained 5 µL of vRNA, Superscript III Platinum One-step qRRT-PCR reaction mix (Invitrogen), 0.5 µL of ROX (internal reference dye) as a passive reference, 0.2 µM probe, and 0.4 µM of each primer. The following thermal profile was used on an ABI Prism 7000 SDS Real-Time apparatus (Applied Biosystems): 30 min at 45°C for reverse transcription; 2 min at 95°C to inactivate the reverse transcriptase and activate the DNA polymerase; and then 40 amplification cycles of 15 s at 95°C and 1 min at 60°C each. Triplicates of negative samples were included in each experiment. Each fluorescent reporter signal was measured against the ROX signal to normalize for non-PCR–related fluorescence fluctuations between samples. Data were collected at the annealing step of each cycle, and the threshold cycle (Ct) for each sample was calculated by determining the point at which the fluorescence reached the threshold limit. Concentration of unknown samples was determined using a standard curve based on the Ct values and the amount of RNA standards.

**Figure 3:**
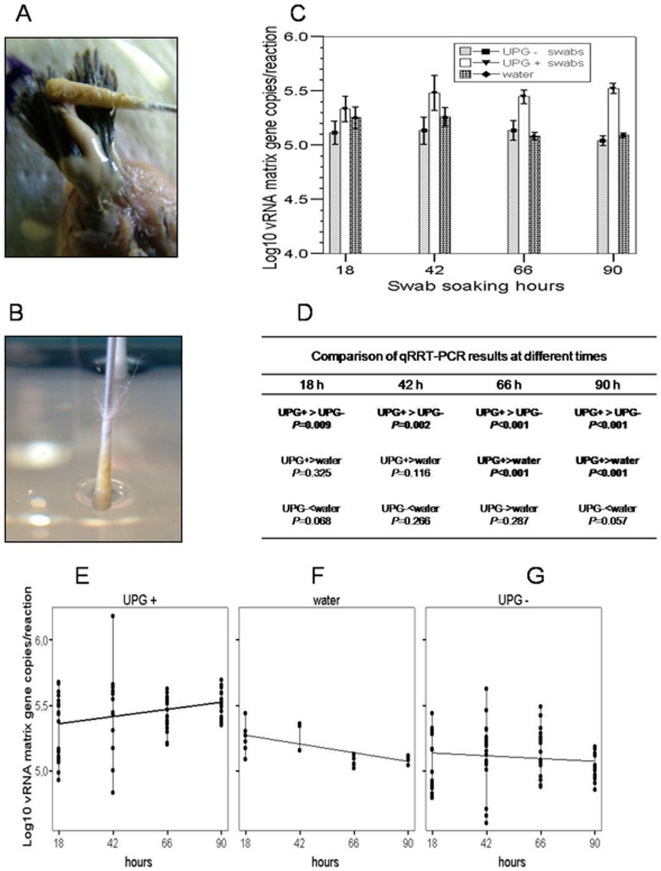
AIV capture from AIV-contaminated fresh water and concentration on preen oil impregnated swabs. (A) Swabs impregnation with preen oil (UPG+). (B) Swabs soaked in AIV-contaminated water. (C) Bar graph representing mean ±2 SEMs of log_10_ of vRNA M gene copies per reaction calculated at each interval time from 3 preened (UPG+) swabs and 3 unpreened (UPG–) swabs that were soaked in AIV-contaminated fresh water; the contaminated fresh water samples were used for comparison. (D) Pair comparison of qRRT-PCR results. Bold type indicates significant differences calculated by 2-tailed Student's t-test. (E–G) Relationship between log_10_ of vRNA M gene copies detected in (E) UPG+ swabs with respect to swab-soaking hours, (F) water with respect to water-collection hours, (G) UPG– swabs with respect to swab soaking hours. UPG+ and UPG–: Swabs impregnated with preen oil and non-impregnated Swabs, respectively.

Because all cover feathers of AIV–reservoir birds are naturally preened, we conducted an experiment in which preen oil–impregnated swabs and preen oil–free swabs were used to determine whether uropygial secretion induces virus adsorption and concentration. In this experiment (experiment 2) fresh river water (12 L, pH 7.5, salinity 0 ppt, density 1000 kg/m^3^, previously tested negative for AIVs) was inoculated with the same AIV strain and concentration that was used in experiment 1. Twelve cotton swabs impregnated with AIV-negative uropygial gland secretion (UPG+) (Figure 3A) and 12 control cotton swabs without any secretion (UPG-) were soaked in AIV-infected water at 10°C (Figure 3B). After 18, 42, 66, and 90 h post-exposure, 3 swabs of each type were placed in transport media (500 µL), vortexed for 5 min, incubated for 12 h at 4°C, and underwent a recovery process (after 5 min of vortexing, each swab was centrifuged upside down at 200×g for 10 min), resulting in a final volume of approximately 550 µL per tube. At each time interval, a separate water sample collected at the same water level served as a baseline to compare viral concentration in the swabs.

Samples of vRNA were extracted from all processed transport media and water samples (stored at −80°C) and analyzed by qRRT-PCR to quantify M-gene copies as described for experiment 1. For both experiments, 2 different qRRT-PCR assays were performed, and each sample was tested in triplicate. Water samples from experiment 1 were tested for a third time in triplicate.

### Virus Titration

The EID_50_/g of the VI–positive feather sample (field samples) and the virus-contaminated feather tuft (experiment 1) were calculated according to the method of Spearman and Kaber [29]. To calculate the EID_50_/g of the field feather samples, we used our previous estimate of the weight of matrices collected from feathers (i.e., mean values of differences obtained between swabs weighed after and before the rubbing activity on body surface). The EID_50_/g of examined transport media (log_10_ value) was corrected according to the dilution of matrices in transport media (0.002 g in 1 g = 1∶500). The method of Reed and Muench [29] was used to calculate the virus titer of infected allantoic fluids (experiments 1 and 2) and contaminated water (experiment 1).

### Statistical Analyses of Field Results and Real-Time PCR Data

All data were analyzed by SPSS for Windows version 12.0 (SPSS, Inc., Chicago, IL). The significance level was set at α = 0.05. Qualitative data of the field study, expressed as positive or negative results obtained using 345 cloacal swabs and 345 feather swabs examined by molecular and virological methods, were analyzed by the Pearson's chi-square test.

In experiment 1, the Kolmogorov-Smirnov test for goodness of fit was used to verify the normality of the distribution. On the basis of this test (*Z* = 0.529; *P* = 0.943), the Student's t-test was used to compare the log_10_ of quantitative data. Means and standard error of means (SEM) were calculated for qRRT-PCR results, expressed as log_10_ vRNA M gene copies per reaction obtained from UPG+ feather tuft samples (*n* = 5) tested twice in triplicate and virus-contaminated water sample (*n* = 1) tested thrice in triplicate. The parametric Student's t-test (2-tailed) was performed to verify possible differences between the mean of the log_10_ of M gene copies in UPG+ feather tufts and in virus-contaminated water samples at 24 h.

In experiment 2, the Kolmogorov-Smirnov test for goodness of fit was used to verify the normality of the distribution. On the basis of the results of the Kolmogorov-Smirnov test (*Z* = 0.973; *P* = 0.300), the Student's t-test was used to compare the log_10_ of quantitative data. Means and SEMs were calculated for qRRT-PCR results, expressed as log_10_vRNA M gene copies per reaction at 18, 42, 66, and 90 h in UPG+ swabs (*n* = 3), UPG-control swabs (*n* = 3), and virus-contaminated water sample (*n* = 1), tested twice in triplicate. The parametric Student's t-test (2-tailed) was used to compare qRRT-PCR results (mean of log_10_ vRNA M gene copies per reaction) detected in UPG+ swabs (*n* = 3) versus UPG– swabs (*n* = 3); UPG+ swabs (*n* = 3) versus contaminated water (*n* = 1); and UPG– swabs (*n* = 3) versus contaminated water (*n* = 1). Linear regression was used to evaluate the relationship between log_10_ vRNA M gene copies in UPG+ swabs with respect to the number of hours the swab was soaking, between log_10_ of vRNA M gene copies in water with respect to water collection hours, and between log_10_ of vRNA M gene copies in UPG– swabs with respect to the number of hours the swab was soaking.

### Optimization of RT-PCR Assays and preliminary molecular tests

Because we examined different biological samples in the field studies, we tested the diagnostic efficiency of the one-step RT-PCR method used. First, equal quantities of feathers and fecal samples were collected from influenza-negative ducks and suspended in transport media (1/40; w/v). After an 18-h incubation at 8°C to 11°C, aliquots of fecal and feather transport media were prepared and spiked with the LPAIV A/Mallard/Italy/228090/2005 (H5N1) that was added to both aliquots with a final titer of 10^6.6^ EID_50_/mL. These two viral suspensions were diluted 10-fold in the PBS/glycerol transport media up to a virus titer of 10^0.6^ EID_50_/mL. From each 10-fold dilution of aliquots, vRNA was extracted and amplified, as previously described for our field studies.

The same molecular methods were used to exclude AIV presence in uropygial secretions collected from wild ducks. Specifically, we swabbed glands of 100 of the 345 birds trapped in the present study. The salt water, fresh water, and uropygial secretions used in experiments 1 and 2 were previously tested by qRRT-PCR (see Laboratory Experiments to Detect and Quantify AIVs).

## Results

### Field Studies: Detection of AIVs on Wild Mallards' Feathers

To better analyze the field results, we categorized ducks into 3 groups on the basis of results obtained by submitting feather and cloacal swabs to RT-PCR (Figure 1B) and VI (Figure 1C): (1) AIV-positive from feather swabs only (F group); (2) AIV-positive from cloacal swabs only (C group); and (3) AIV-positive from both feather and cloacal swabs (F and C group).

Results from one-step RT-PCR showed that 27% of ducks from the F and from F of F and C groups were AIV-positive on feathers. Statistical analysis of RT-PCR data (Figure 1B) showed more RT-PCR–detection of AIV from feathers (93/345) than from cloacal swabs (38/345) (Pearson's chi-square test = 28.503; *P*<0.001). When RT-PCR–positive samples were inoculated into embryonated eggs, 16/345 ducks were AIV-positive from feather swabs and 15/345, from cloacal swabs (Figure 1C). Statistical analysis of data from RT-PCR–positive samples tested for VI showed higher VI percentage in cloaca-positive ducks (15/38) than in feather-positive ducks (16/93) (Pearson's chi-square test = 7.406; *P* = 0.007). The 4.3% (15/345) prevalence of VI in cloaca-positive Mallards agrees with results of previous studies in the same Mediterranean wintering area [30],[31].

Virus characterization by serologic and sequence analyses showed that all isolates were LPAIVs belonging to the H12, H10 (most prevalent), H9, H8, H5, H4, and H3 subtypes. To determine the AIV infectivity on the birds' body surfaces, we calculated the virus titer from 1 of 16 VI–positive feather swabs collected in this study and expressed as 10^4.6^ EID_50_/g. By preliminary spiking assays used to test the diagnostic efficiency of RT-PCR, both feather and cloacal samples showed the same positive threshold value (10^2.6^ EID_50_/100 µL). Finally, the initial presence of AIVs in secretions of the uropygial gland in wild ducks was excluded. Uropygial secretions from all 100 birds were AIV-negative by RT-PCR, although 7 ducks were AIV-positive on the feather surface.

### AIVs Interact with Preened Feathers and Swabs in Water

In experiment 1, significant differences (Student's t-test = −6.242; *P*<0.001) were found between the mean of log_10_ values of M gene copies in UPG+ feather tufts (5.26) and that in salt water (4.54). Figure 2C shows mean ±2 SEMs of log_10_ of vRNA M gene copies per reaction calculated after 24 h by qRRT-PCR on 5 preened (UPG+) feather tufts and AIV-contaminated water. Moreover, viral concentration of each feather sample was significantly higher than that of the water control (Two-tailed Student's t-test; *P*<0.001). The differences in log_10_ M gene copies in preened feathers may be due to varying dimensions of feather tufts as well as natural or intrinsic experimental variability. At the end of experiment 1, virus titers of contaminated water and that of impregnated feathers were 10^3.6^ EID_50_/mL and 10^4.1^ EID_50_/g, respectively, showing an increase of 0.5 log_10_ after 24 h.

In experiment 2, M gene copies for UPG+ swabs were higher than those for UPG– swabs at all time intervals, whereas M gene copies for UPG+ swabs were significantly higher than those for control water at 66 and 90 h only (Figure 3D). There was no significant difference in M gene copies for UPG– swabs and control water. Figure 3C shows the mean ±2 SEMs of vRNA M gene copies per reaction (log_10_) at each examination time in UPG+ swabs, UPG– swabs, and control water. Differences in M gene copies in UPG+ swabs (Figure 3E), control water (Figure 3F), and UPG– swabs (Figure 3G) followed different trends. UPG+ swabs progressively concentrated and increased AIV amounts (Pearson's correlation coefficient *r* = 0.285; *P* = 0.018). This increase occurred while the concentration of virus in the water decreased with time (*r* = −0.647; *P* = 0.001), the viral concentration in UPG– control swabs appeared to decrease, though the change was not significant (*r* = −0.119; *P* = 0.322).

## Discussion

Our results suggest there may be a previously unrecognized concentration mechanism of AIVs on the bodies of aquatic birds and a potential preening-mediated route of infection. Virus adsorption on bird bodies appears to be a natural mechanism by which virus particles are captured by preened feathers and concentrated from the aquatic environment to bird bodies. Our findings indicate that a progressive virus “sticking” occurs because AIV-contaminated waters interact with the uropygial gland secretion, which covers body surfaces. By preening, birds spread preen oil all over their plumage, and this behavior could facilitate a protracted ingestion of AIV particles, thus possibly promoting a preening-mediated infection. In natural conditions, mallards spend 10.9% of their daily time engaged in grooming behavior [32],[33], including preening activities which are necessary for waterproofing, heat regulation, and supplying provitamin D by preen oil–ingestion [11],[34]. In such a context, self-preening, allopreening, or both could improve the efficiency of the indirect water-borne transmission route.

Results from our field studies demonstrate a consistent presence of viral RNA and infectious AIVs on birds' bodies. In particular, we showed that in 345 free-living Mallards that tested positive for AIVs by RT-PCR, feather swabs were 2.5 times more often AIV-positive than cloacal swabs (27% *vs* 11%), whereas VI percentages (calculated for RT-PCR–positive birds only, to compare virus infectivity of 38 cloacal samples and 93 feather samples) were 2.3 times higher in cloacal swabs than in feather swabs (39.5% *vs* 17.2%). These significant differences suggest partial inactivation of AIVs stuck on feathers, probably due to environmental factors such as UV rays or unsuitable temperatures. The absence of AIVs in uropygial glands of wild birds suggests the external origin of AIVs detected on the ducks' surface and supports our hypothesis that preened bodies are an ecologic link between aquatic birds and the environmental persistence of AIVs. Additionally, the results of experiments 1 and 2 demonstrate that preen oil can capture and concentrate virus particles added to either salt water or fresh field water. The capture of AIV from experimentally contaminated waters by feathers and swabs impregnated with preen oil strongly suggests that virus particles accumulate on preened feathers in natural environments.

If the proposed preening-mediated mechanism of infection is at play in nature, birds carrying viruses on their feathers but testing negative for virus in the cloaca and trachea by current surveillance programs [35] might still play an active role in spreading AIV infection. These “false-negative” birds could include susceptible birds that are naïve to AIV infection, as well as unsusceptible birds that are naturally immunized to AIV infection. In the second case, this novel infection mechanism might escape the birds' immune system [36],[37] such that unsusceptible hosts might infect susceptible birds by allopreening.

Results from our field studies indicate that AIVs can be carried on the feather surface of infected ducks (i.e., those VI-positive from both cloacal and feathers swabs) and uninfected ones (i.e., those VI-positive from feathers only). For this reason, in routine surveillance programs, additional sampling methods could be necessary to detect AIVs on birds' bodies. Our field and experimental results also suggest that during the time period between the virus adhesion to the bird's body and the infection (possibly due to self- and/or allopreening), the virus could move in nature with the host by an undescribed circulation mechanism. In such a context, the epidemiologic status of uninfected birds carrying AIVs on their feathers certainly does not affect the fitness of the host, in contrast to what is reported for LPAIV [38],[39] and HPAIV [40] infections. With particular regard to the geographical spread of the Eurasian H5N1 HPAI virus in wild birds, the uninfected carrier hosts could have facilitate, by preening behavior, the circulation of a virus able to kill the natural reservoir [41]–[47].

The presence of Eurasian H5N1 HPAI virus on swan feathers, possibly due to the preen oil–virus interaction or fecal contamination, may also explain the only recorded human case of fatal infection passed from wild birds in February 2006 [48]. All infected humans were involved in defeathering of dead wild swans after a massive die-off of these aquatic birds occurred in Azerbaijan. Because women defeather birds more often than men do, their high exposure to infected feathers may explain their higher incidence of infection [49].

Our study differs from previous reports on Eurasian H5N1 HPAI virus in domestic duck feathers because we examined mature, rather than growing, feathers. The H5N1 HPAIV is in feathers from young (2- and 4-week-old) Mallards experimentally inoculated with H5N1 HPAIV [50]-[53]. In this age group, birds are unable to fly, and growing feathers are living tissue; therefore, detection of H5N1 HPAI virus inside duck calamuses was because of viremia or virus replication [53]. In contrast, swabs in our study were taken from feathers on the external body surface of ducks sampled after the moult, when only mature feathers, which are dead tissue [54], cover the body. Thus, we substantiate that AIVs detected on birds with mature feathers originate from the external environment.

Our results also suggest that a preened body surface could be the common denominator that explains how AIV infection occurs in different taxonomic groups of aquatic birds. Because the chemical composition of preen oil changes with the season as well as across species and ages of birds [55],[56], future studies are needed to determine the common uropygial component that could promote interaction with AIVs in all aquatic bird species.

Our newly proposed mode of AIV circulation in aquatic birds integrates well with the recently proposed epidemiologic approach that emphasizes the role of environmental transmission of AIVs [57]–[59]. The preening-mediated infection mechanism could be implicated in AIV dispersal in nature, but additional work is required to determine how this mechanism could affect the long-distance movements and long-term infectivity of AIVs. Natural variables related to the virus (i.e., seasonal prevalence, viral load, and environmental persistence) and host biology (i.e., flock size, population density, migratory behavior, and moulting period) are at play in the wide context of influenza ecology [60], and their potential effects on the mechanism proposed here are unclear. However we believe that these findings expand our knowledge of AIV ecology.

## Supporting Information

Figure S1 Flow chart of virologic assays conducted to test feather and cloacal swabs collected from mallards. The symbol (X) indicates the stage when the analysis was stopped. However, the initially collected samples of all available RT-PCR-positive swabs, even those from which virus could not be isolated, were used to inoculate embryonated eggs again (*).
(0.04 MB DOC)
