# Supplementary material for: Can Preening Contribute to Influenza A Virus Infection in Wild Waterbirds?
Source: PLoS One. 2010 Jun 25;5(6):e11315. doi: 10.1371/journal.pone.0011315 (PMC2892510; doi:10.1371/journal.pone.0011315)
Supplement: Figure S1 — Flow chart of virologic assays conducted to test feather and cloacal swabs collected from mallards. The symbol (X) indicates the stage when the analysis was stopped. However, the initially collected samples of all available RT-PCR-positive swabs, even those from which virus could not be isolated, were used to inoculate embryonated eggs again (*). (0.04 MB DOC) [file pone.0011315.s001.doc]

**SAMPLES FROM WILD DUCKS**

Pool of 5 cloacal swabs (or pool of 5 feathers swabs) was screened by RT-PCR

Pool of 5 cloacal swabs (or pool of 5 feathers swabs) was screened by RT-PCR

Negative pool

If the pool was positive, the RT-PCR analysis was further carried out on single swabs

Single swab was analyzed by RT-PCR

If a single swab was positive by RT-PCR the analysis was carried out by virus isolation assay

Negative single swab

Inoculation of swab transport medium in SPF embryonated chicken eggs (1^st^ passage)

HA-positive

HA-negative

Hemagglutination inhibition (HI) test and/or molecular characterization by subtype-specific RT-PCR and sequencing

ELISA test for influenza A virus nucleoprotein on allantoic fluid

Inoculation of allantoic fluid in SPF embryonated chicken eggs (2^nd^ passage)

ELISA Positive

ELISA negative

ELISA test for influenza A virus nucleoprotein on allantoic fluid

ELISA negative

ELISA Positive

*****
